# Retraction: Improved support vector machine based on CNN-SVD for vision-threatening diabetic retinopathy detection and classification

**DOI:** 10.1371/journal.pone.0332845

**Published:** 2025-09-22

**Authors:** The PLOS One Editors

The *PLOS One* Editors retract this article [1] due to concerns about:

- The article’s compliance with PLOS policies on Data Availability and PLOS Policies on Materials, Software and Code Sharing.
- The article’s adherence to the journal’s third and fourth publication criteria [2].

The first author provided the available quantitative data underlying the results of [1], but stated that the data underlying Fig 13 are no longer available. In addition, the first author submitted the available underlying code files to [3]. They stated that the code files underlying the CNN-SVD feature extraction are no longer available. During editorial follow up the first author provided contradicting statements regarding the availability of the underlying ISVM-RBF code files. A member of the *PLOS One* Editorial Board reviewed the available underlying code files and raised concerns that these did not reflect the core innovations in the article and the available files had limited reusability and maintainability. They commented that the level of detail in the article and the available underlying code files was insufficient to replicate the methods and results in [1].

In addition to the above concerns, the *PLOS One* Editors identified multiple citation errors in this article [1] that were not resolved during editorial follow-up with the authors. Additionally, after this article [1] was published, concerns were identified about the peer review.

PLOS regrets that the issues were not addressed prior to the article’s publication.

AB, AI, TIB, AA, and MS did not agree with the retraction. XL and HL either did not respond directly or could not be reached.

Fig 2 and Figs 4–6 contain images obtained from the iDriD database [4] which are offered under a CC BY 4.0 license.
